# Resistant Traits in Digital Organisms Do Not Revert Preselection Status despite Extended Deselection: Implications to Microbial Antibiotics Resistance

**DOI:** 10.1155/2014/648389

**Published:** 2014-05-20

**Authors:** Clarence F. G. Castillo, Maurice H. T. Ling

**Affiliations:** ^1^School of Information Technology, Republic Polytechnic, Singapore 738964; ^2^School of Chemical and Biomedical Engineering, Nanyang Technological University, Singapore 637459; ^3^Department of Zoology, The University of Melbourne, VIC 3010, Australia

## Abstract

Antibiotics resistance is a serious biomedical issue as formally susceptible organisms gain resistance under its selective pressure. There have been contradictory results regarding the prevalence of resistance following withdrawal and disuse of the specific antibiotics. Here, we use experimental evolution in “digital organisms” to examine the rate of gain and loss of resistance under the assumption that there is no fitness cost for maintaining resistance. Our results show that selective pressure is likely to result in maximum resistance with respect to the selective pressure. During deselection as a result of disuse of the specific antibiotics, a large initial loss and prolonged stabilization of resistance are observed, but resistance is not lost to the stage of preselection. This suggests that a pool of partial persists organisms persist long after withdrawal of selective pressure at a relatively constant proportion. Hence, contradictory results regarding the prevalence of resistance following withdrawal and disuse of the specific antibiotics may be a statistical variation about constant proportion. Our results also show that subsequent reintroduction of the same selective pressure results in rapid regain of maximal resistance. Thus, our simulation results suggest that complete elimination of specific antibiotics resistance is unlikely after the disuse of antibiotics once a resistant pool of microorganisms has been established.

## 1. Introduction

Many microorganisms, such as* Streptomyces*, naturally secrete chemical agents that are toxic to other microorganisms as a self-defense mechanism and had been exploited for medical use as antibiotics [1]. However, many pathogenic microorganisms are developing resistance to currently available antibiotics [2] as it has been shown that the prevalence of resistant strains is significantly higher in areas of antibiotics use compared to areas without antibiotics use [3, 4]. Some of the antibiotics resistance mechanisms employed by microorganisms include modifying cell wall architecture [5], actively pumping antibiotics out of the cell [6], and mutating the protein molecules targeted by antibiotics to reduce binding efficiencies [7]. Many microorganisms found in the intestinal tract are exposed to sublethal concentrations of oral antibiotics or dietary chemicals [8] as a result of incomplete absorption, which subsequently leads to resistance or tolerance development in intestinal microorganisms [9–11]. This is supported by a study in which pigs are treated with ampicillin, a common antibiotic, and demonstrates a significant increase in the occurrence of ampicillin-resistant* Escherichia coli* (a common intestinal bacterium) from 6% to more than 90% after a course of 7 days [12]. A study had found that the low persistence of antibiotics resistance in intestinal bacterium can be found 12 years after disuse of the antibiotics [13].

As chemical resistance (such as antibiotics resistance) is a result of selective pressure in the presence of the chemical, it can be expected that the prevalence of resistance will decrease following withdrawal and disuse of the chemical. An early study by Peffly and Shawarby [14] demonstrates that the toxicity of houseflies to insecticide reverts to near pretreatment levels after cessation of insecticide application. A study in France shows that reduction of antibiotics use correlates with the reduction of microbial resistance [15]. Although similar reduction of microbial resistance after disuse had been reported in other parts of the world [16], a number of studies have reported contradictory results [13]. For example, Enne et al. [17] report the persistence of resistant* E. coli* 8 years after reduction of antibiotics use. This suggests that the loss of chemical resistance following disuse, after the acquisition of chemical resistance traits under selective pressure exerted by the presence of the chemical, is not straightforward [13]. However, examining the acquisition-loss-reacquisition of resistance traits is difficult to perform experimentally as it will require extensive sequencing of individual organisms or bacterium as each organism may be genetically different from the other during the process. Hence, such an experimental study will be expensive.

In this study, we use DOSE [18], a digital evolution platform containing self-replicating “digital organisms” (DOs), to examine the acquisition-loss-reacquisition of resistant traits as fitness. This is under the assumption that there is no fitness cost for maintaining resistance. DOs offer the advantage of ability to examine each genome and had been successfully used in many evolutionary studies [19, 20]. We hypothesized that fitness will be gained under selective pressure and such fitness will decline in subsequent removal of selective pressure. Our results show that selective pressure is likely to result in resistance with respect to the selective pressure and a pool of partial resistant organisms is likely to persist long after withdrawal of selective pressure. Subsequent reintroduction of the same selective pressure results in rapid regain of resistance, suggesting that complete elimination of specific antibiotics resistance is unlikely after the disuse of antibiotics, once a resistant pool of microorganisms has been established.

## 2. Methods

*DOSE Platform*. The details of DOSE (digital organism simulation environment) had been described [18, 21–23]. Briefly, a population comprises of one or more DOs, which is made up of a genome and a set of statuses. The genome can comprise one or more chromosomes of varied length. Although there is no restriction to the set of nucleotides, common nucleotide sets are binary, integer, and alphanumeric. A background mutation rate is set for each chromosome and an additional mutation rate can be set during the simulation. In addition, specific mutations on a base can be made. The genome can be expressed virtually by the Ragaraja interpreter [23], which is a Turing machine that uses an integer chromosome as code. This results in executable DNA similar to that of Avida [24]. DO statuses consist of traceable information regarding the organism (such as identity), as well as vital statuses (such as age). One or more populations can reside in the world, which comprises one or more ecological cells arranged in one or more dimensions. During simulation, 6 simulation-dependent operations are executed on each population. Firstly, premating population control can be used to remove organisms of low vitality. Secondly, execute mutation scheme. Thirdly, measuring fitness before and after execution of mating scheme can include a range of mutational operations, such as point mutations, deletions, inversion, duplication, and translocation. Fourthly, postmating population control can be used to remove organisms of low fitness. Fifthly, execute ad hoc generational events, which can be used to simulate ad hoc events such as radiation bursts. Lastly, move organisms to another ecological cell if required.

*Simulation Setup*. One population consisting of a hundred DOs, each consisting of a chromosome of 500 binary nucleotides as genome, was used for simulation. The ancestral chromosome is set as 500 bases of alternate binaries, that is, [1010101010]_50_. Background mutation rate is set at 1% or 5 base mutations per generation and using only point mutation. All DOs were deployed within one ecological cell.

*Experiment  1 *(initial gain of resistant trait). Gain of resistance as a result of selective pressure on microorganisms as a result of antimicrobial chemical use was simulated by selecting for desired resistance. A predetermined nucleotide sequence was defined as a representation of the desired resistance trait, where complete and partial fulfillments of the predetermined sequence were defined as complete and partial resistance, respectively. Four different sequences were used to examine different complexities of resistant traits—10 nonconsecutive blocks of 5, 7, 9, and 11 zeros. Fitness score for each DO was calculated as the sum of the number of consecutive zeros within a block for 10 blocks. The following rules were used. Firstly, at least 2 consecutive zeros were required to be considered a block. For example, “101101011” would result in a fitness score of 0, while “101001011” would result in a fitness score of 2 as there were at least 2 consecutive zeros. Secondly, a block would only contribute to 10% of the maximum fitness score. For example, if the predetermined sequence was 10 nonconsecutive blocks of 5, thereby, the maximum fitness score is 50. Each block will have a maximum fitness score of 5. This implied that “101000011,” “100000111,” and “100000011” would result in fitness scores of 4, 5, and 5, respectively. Thirdly, a maximum of 10 blocks contributed to the fitness score. In each generation, all DOs will undergo random mutations and replicate, resulting in 200 DOs. Two different selection schemes were tested. In truncation selection (TS), the top 100 fittest DOs will be retained for the next generation. In fitness-proportionate selection (FPS), 100 DOs were selected where the probability of a DO being selected is directly proportional to its fitness score. 25 replicates of 200 generations were simulated for each of the 4 resistance sequences and the fitness scores of each of the 100 postculled DOs were recorded. 

*Experiment  2 *(loss of resistant trait). Each population at the 200th generation (corresponding to the last generation of initial gain of resistance experiment) was revived and simulation continued for another 5000 generations for loss of resistant trait; thus, giving a final generation count of 5200. In these 5000 generations, culling of population after replicate would be random and not affected by fitness scores of the DOs to simulate the withdrawal of selective pressure from the use of antimicrobial chemicals. The fitness scores of each of the 100 postculled DOs were recorded.

*Experiment  3 *(regain of resistant trait). Regain of resistant trait as a result of reintroduction of antimicrobial chemicals after extended periods of disuse was simulated by revival of each population at the 5200th generation (corresponding to the last generation of loss of resistance experiment) and simulation continued for another 200 generations. The simulation processes (population culling using fitness scores) of these 200 generations were identical to that of Experiment*  *1 (initial gain of resistant trait) to simulate reintroduction of antimicrobial chemicals.

*Experiment  4 *(repeated loss and regain of resistant trait). Repeated loss and regain of resistant trait was carried out on one resistance sequence—10 blocks of 11 zeros—to simulate multiple cycles of reintroduction and disuse of antimicrobial agents. Populations for 10 blocks of 11 zeros were revived from the 5200th generation for 5000 generations of resistance loss. The simulation of resistance loss was identical to that of Experiment*  *2 (loss of resistant trait). This cycle of repeated gain and loss of resistance trait was repeated for 3 additional cycles, resulting in a total of 26000 generations. The average fitness scores of each successive resistance gain (generations 5201–5400 as Gain-2, generations 10401–10600 as Gain-3, generations 15601–15800 as Gain-4, and generations 20801–21000 as Gain-5) were analyzed.

## 3. Results

**Figure 1:**
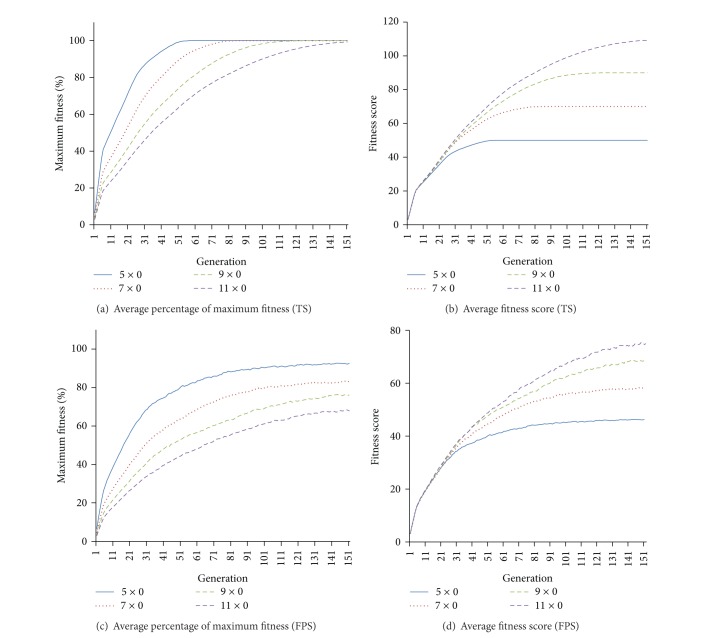
Gain of resistant traits under selective pressure. Panels (a) and (b) are results from TS, while Panels (c) and (d) are results from FPS. Panels (a) and (c) show the average (*n* = 25) percentage of maximum fitness scores across 200 generations for the 4 different complexities of resistant traits, referred to as “target sequence,” from truncation selection and fitness-proportionate selection, respectively. For example, “target sequence = 5 × 0” refers to the simulation of 10 blocks of 5 zeros as resistant trait. Panels (b) and (d) show the average absolute fitness scores from truncation selection and fitness-proportionate selection, respectively.

**Figure 2:**
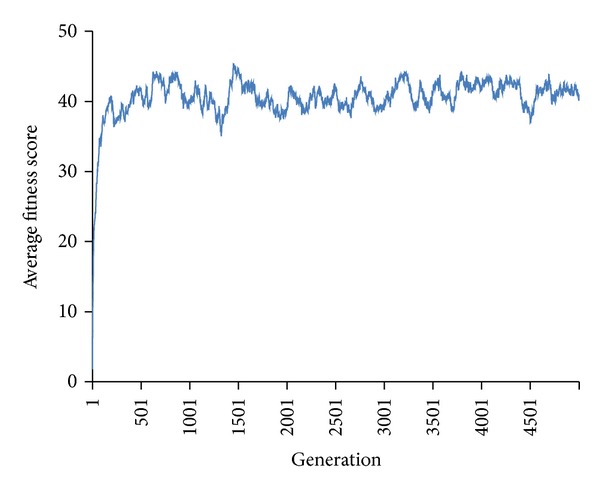
Fitness in unselected population across 5000 generations as control.

*Experiment  1 *(initial gain of resistant trait). As hypothesized, all simulations demonstrated an increase in average population fitness and percentage of DOs achieving maximum fitness regardless of the selection methods used (truncated selection or fitness-proportionate selection) and the complexity of various fitness requirements (Figure 1) within 200 generations. The rate of fitness gain, in terms of percentage of maximum fitness, is dependent on the complexity of the resistant traits. Although fitness gain is observed in every case regardless of the use of truncation selection (TS) or fitness-proportionate selection (FPS), only the use of TS is able to achieve maximum fitness within 200 generations. Despite that, more than half of the population in FPS achieved maximum fitness within 200 generations and there is no obvious difference in the initial rate of absolute average fitness scores gained (Figures 1(b) and 1(d)) from 25 replicated simulations. Under no selective pressure, which acts as control, the average fitness score is 40.6 with a standard deviation of 2.76 (Figure 2) across 5000 generations. By comparing between the presence and absence of selective pressure, our results show that the fitness scores under selective pressure are significantly higher (paired *t*-test *P* value < 4.2 × 10^−90^ for TS and paired *t*-test *P* value < 2.2 × 10^−90^ for FPS from generation 1 to 200) than that of no selective pressure in spite of lower average population fitness and the percentage of organisms achieving maximum fitness in FPS compared to TS. 

**Figure 3:**
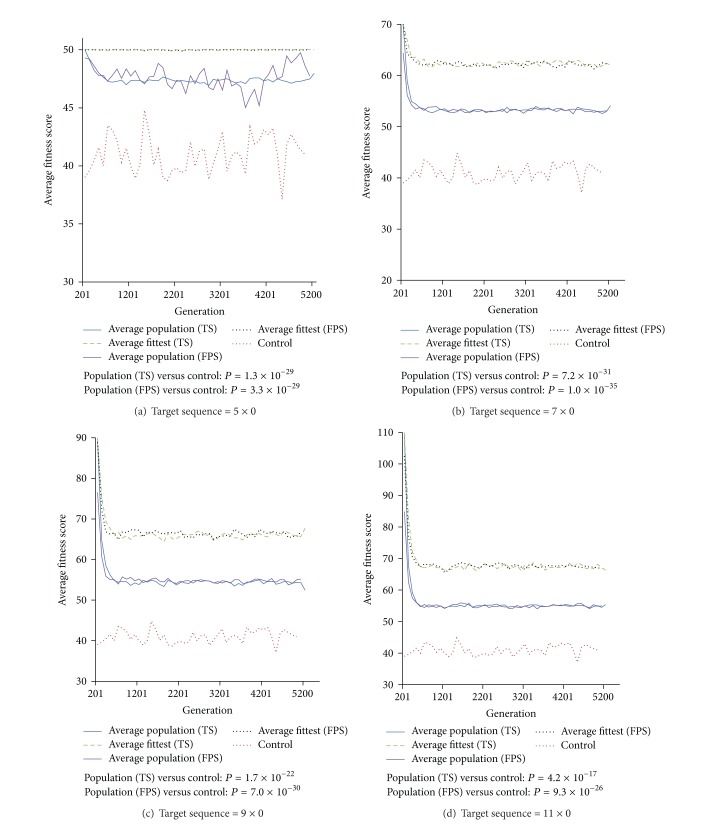
Average fitness score for 5000 generations of deselection (generation 201 to 5200). Panels (a), (b), (c), and (d) show 4 different resistant complexities (referred to as “target sequence”), respectively. The average population fitness and the average fitness of the fittest organism of each triplicated simulation are shown for each generation. For each target sequence, the corresponding average fitness scores at each generation for control (from Figure 2) are added for comparison. Hence, the average fitness scores for controls in each panel are identical. Paired *t*-tests are performed between average population fitness and control for both TS and FPS.

*Experiment  2 *(loss of resistant trait). All populations of both selection methods are subjected to 5000 generations of selective pressure withdrawal to simulate the disuse of specific antibiotics after prevalence of resistance. Our results show that there is an initial decline of fitness before plateauing following deselection in every scenario (Figure 3). By comparing the average population fitness between TS and FPS and the average fitness score of the fittest organism between TS and FPS, our results do not show significant differences in the fitness (paired *t*-test *P* value > 0.08). This suggests that the effect of different selection methods is not carried forward to affect deselection.

Comparing with the average fitness of control populations (no prior selective pressure), the average population fitness at plateau following deselecton is significantly higher than control (paired *t*-test *P* value < 4.2 × 10^−17^) in every deselection. However, the results suggest a wide range of fitness within the population. Our results demonstrate significant difference (paired *t*-test *P* value < 2.7 × 10^−21^) between the average fitness score from 25 replicated simulations of the fittest organism and the average population fitness. This suggests that a pool of partial resistance is established in each case despite an initial decline of average population fitness following deselection.

Regression analyses were performed on the average population fitness and average top fitness from generation 2000 to 5200 for each case (Table 1). This examines the likelihood of the fitness and the expected number of generations required for deselection process in order for the average population fitness to decline to the fitness without prior selection, which can be estimated by the average population fitness of the control. As the selection methods do not result in significant differences in the average fitness during deselection, only data from FPS were used for regression analysis. Our results show that the gradient of fitness change across generations is not statistically significant at 95% confidence in every selection scenario. This implies that the average fitness may be constant, decrease, or even increase during deselection.

**Figure 4:**
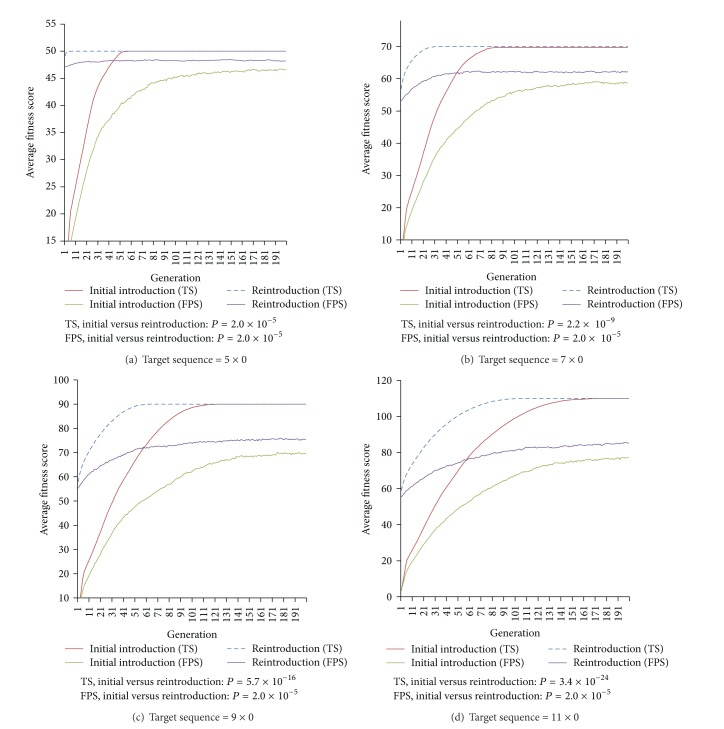
Average population fitness score for 200 generations of reintroduction of selective pressure. Reintroduction of selective pressure was carried out from deselection experiment (generation 5200 in Experiment 3). This is compared to initial introduction of selection to a native population (Experiment 1) and paired *t*-test is performed between the generation-matched average fitness of initial introduction (generation 1 to 200) and reintroduction (generation 5201 to 5400) for both TS and FPS.

*Experiment  3 *(regain of resistant trait). All populations at the end of Experiment*  *2 (generation 5200) are subjected to 200 generations of reintroduction of selective pressure to examine the likely rate of reemergence of the same phenotypic antibiotics resistance if previously used antibiotics are reused following an extended disuse as a result of former prevalence of resistant strains. Our results are consistent with that of Experiment*  *1, showing that the average fitness scores in TS are higher than that of FPS. Despite that, our results show that reemergence of antibiotics resistance is significantly faster (paired *t*-test *P* value < 2.0 × 10^−5^) at the reintroduction of selective pressure in every scenario (Figure 4). This suggests that reintroduction of selective pressure results in faster reemergence of antibiotics resistance regardless of selection methods.

**Figure 5:**
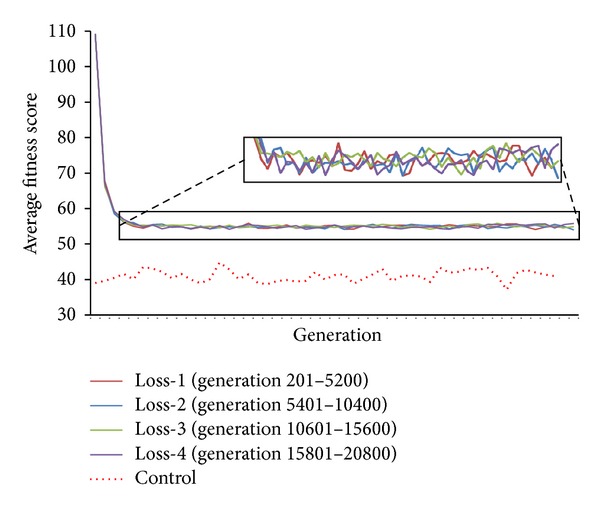
Average population fitness in 4 consecutive TS deselections. There is no significant difference between consecutive deselections and between TS and FPS deselections (see Table 2; paired *t*-test *P* value > 0.15).

*Experiment  4 *(repeated loss and regain of resistant trait). We repeated the loss and gain of resistant trait to examine the effects of repeated reintroduction of antibiotics as selective pressure. Our results from 4 deselections show that initial loss of population resistance, measured by fitness, occurs in the early deselection before plateauing. Statistical analysis from pairwise paired *t*-test between the 4 deselections suggests that the loss of resistance trait does not vary significantly between any 2 deselections (Table 2; *P* value from pairwise paired *t*-test for TS > 0.06 and *P* value from pairwise paired *t*-test for FPS > 0.45) but is significantly different between deselections and control (paired *t*-test *P* value < 2.2 × 10^−17^). This suggests that the rate of initial loss of resistance trait and plateauing from the initial loss of resistance trait are similar in every cycle (Figure 5) and unlikely that repeated selection and deselection will revert the population fitness to before initial selection (control). Consistent with the results from Experiment*  *2, there is no significant difference between the decline and plateauing of average population fitness between TS and FPS (paired *t*-test between TS and FPS generations 5401 to 10400 = 0.278, paired *t*-test between TS and FPS generations 10601 to 15600 = 0.156, and paired *t*-test between TS and FPS generations 15801 to 20800 = 0.322).

## 4. Discussion

Antibiotics resistance is an increasing medical issue as a result of prevalent antibiotics use [25]. Hicks et al. [3] and Skalet et al. [4] show that the prevalence of resistant strains is significantly higher in areas of antibiotics use compared to areas without antibiotics use. This is expected as antibiotics represent a selective pressure favouring increasingly resistant strains. Hence, it can be expected that the prevalence of resistance will decrease following withdrawal and disuse of the chemical. However, Enne et al. [17] report the persistence of resistant* E. coli* 8 years after reduction of antibiotics use. As studying the gain and loss of resistance traits experimentally is difficult and expensive, we use DOs as a proxy to study the gain and loss of resistance traits.

We examine two selection criteria in this study—truncation selection (TS) and fitness-proportionate selection (FPS)—as these have been shown to result in different population dynamics [26]. Therapeutic doses of antibiotics are measured as TS [27], in the form of MIC50 and MIC90, which refers to the minimum concentration of antibiotics needed to inhibit 50% and 90% of the microorganisms, respectively [28]. Hence, removing the lower 50% of the population by fitness can be viewed as MIC50. However, FPS has also been used in DOs for evolutionary studies [29] where the probability of an organism selected for the next generation is proportional to the fitness of the organism in the population. Hence, FPS can be seen as a less stringent selection compared to TS.

Despite the theoretical differences between TS and FPS, our results are consistent between TS and FPS for each experiment. This suggests that the conclusions drawn from this study are not artifacts as a result of selection methods. Our results show that the average population fitness and average fitness for the fittest organisms are higher in TS compared to FPS. This is expected as TS has a stronger effect on eliminating less fit organisms compared to FPS.

Our experiments show several important trends. Firstly, there is a rapid gain of resistance traits during initial selective pressure exerted by antibiotics use, and the average population resistance drastically declines following subsequent withdrawal of selective pressure. This is consistent with current studies showing a reduction of resistance after antibiotics disuse [15].

Secondly, the average population resistance after deselection is significantly higher than preselection. The average highest resistance is also significantly higher than the average population resistance despite at least 25 times the length of time for deselection compared to selection (5000 deselective generations versus 200 selective generations). This suggests that a pool of partial or reduced resistant strains may persist long after antibiotics disuse, which is consistent with Enne et al. [17] reporting the persistence of resistant* E. coli* 8 years after reduction of antibiotics use. Using regression analysis, our results suggest that the prevalence of resistance strains remains constant after initial reduction of resistance. This implies that it may be possible for the prevalence of resistance strains to increase even after the disuse of antibiotics at 95% confidence. This situation has been reported in sulfonamide-resistant* E. coli* in United Kingdom where 6.2% increase in the frequency of sulfonamide resistance follows 98% reduction of sulfonamide prescription [17] with no further reduction 5 years after study [30]. This is contradictory to other studies reporting reduction of microbial resistance after antibiotics disuse [15, 16].

Our simulation results suggest that such contradictory results [16, 30] are statistical variations of resistance traits prevalence in long-term disuse of antibiotics. Our findings are consistent with current studies. It is long considered that mutations leading to antibiotics resistance will incur a fitness cost [31], thereby reversing resistance after disuse of antibiotics [32]. Although this has been shown in glycopeptide-resistant enterococci [13] and amphotericin B-resistant* Candida albicans* [33], this phenomenon is not universal as Dutta [31] has shown that secondary mutations on resistant* Salmonella typhi* can outcompete susceptible* Salmonella typhi* strains. A report by Knight et al. [34] also suggests that antibiotics resistance genes may not incur fitness cost in methicillin-resistant* Staphylococcus aureus* (MRSA). On the other hand, Sun et al. [35] report that 8 out of 11 clinical isolates of beta-lactam-resistant* Pseudomonas aeruginosa* show lower growth rate than susceptible strains, which also implies that the remaining 3 out of 11 resistant strains have equal or higher growth rate than susceptible strains. Our simulation experiments do not incur fitness cost on the carrying of resistance traits as random elimination of half of the population after replication is employed. Taken together, this suggests that disuse of antibiotics, to which microorganisms have developed resistance, is insufficient to confer complete susceptibility to the antibiotics as the burden of resistance may or may not result in enough fitness cost to revert resistant strains back to susceptible strains after antibiotics disuse.

Lastly, in the event whereby resistance does not incur fitness cost as in our simulation experiments, our results suggest that a reintroduction of identical antibiotics to which microorganisms have previously developed resistance is likely to result in a faster rate of complete resistance formation. However, this has not been shown experimentally. Theoretically, a possible reason for rapid resistance formation from reintroduction of selection may be the presence of genetic memory of prior events as suggested by Gajardo and Beardmore [36] to be a species preservation strategy by forming resistant structures in some organisms. Another possible reason is proposed by Lee et al. [8] suggesting that resistance to a specific selective pressure may be part of a generic resistance to a range of other selective pressures. The latter has been observed in many microorganisms where a resistance to a specific antimicrobial agent results in cross-resistance to other types of antimicrobial agents [37, 38]. Given that partial resistance persists in the population despite the removal of selective pressure as suggested by our results, it may be plausible that repeated reintroduction of selective pressure may result in increasing partial resistance within the population. However, our simulation results suggest that increasing partial resistance is unlikely as a result of repeated reintroduction of selective pressure alone.

In summary, this study suggests that digital evolution using simulated organisms will be useful to study the effects of selective pressure on population fitness. Our findings suggest that selective pressure as a result of antibiotics or chemical use is likely to cause resistance, which is consistent with previous studies [8, 10, 12] and disuse is unlikely to revert resistant strains back to susceptible strains as a pool of partial resistant strains is likely to persist in the population. More importantly, our results suggest that contradictory reports on the prevalence of resistant strains long after antibiotics disuse [13] may be a result of statistical variation as the pool of partial resistant strains remains constant in the population.

## Figures and Tables

**Table 1:** Estimated number of generations after selection that are needed to lose fitness traits. Regression models are generated from gradual loss of fitness after withdrawal of selection pressure (generation 2000 to 5200) as initial fitness loss (generation 201 to 1999) may overestimate the rate of fitness loss. These regression models were generated using only data from FPS.

	Target sequence	Regression model	Estimated generations needed to lose fitness
Average top fitness	7x0	(−95% CI) Fitness = 62.4 − 0.000017 generation	1,290,000
		(Mean) Fitness = 62.4 − 0.000001 generation	21,800,000
		(+95% CI) Fitness = 62.4 + 0.000017 generation	Infinity
	9x0	(−95% CI) Fitness = 65.5 − 0.000107 generation	233000
		(Mean) Fitness = 65.5 + 0.000119 generation	Infinity
		(+95% CI) Fitness = 65.5 + 0.000345 generation	Infinity
	11x0	(−95% CI) Fitness = 67.3 − 0.000061 generation	438,000
		(Mean) Fitness = 67.3 + 0.000021 generation	Infinity
		(+95% CI) Fitness = 67.3 + 0.000292 generation	Infinity

Average population fitness	5x0	(−95% CI) Fitness = 47.3 − 0.000044 generation	152,000
		(Mean) Fitness = 47.3 + 0.000016 generation	Infinity
		(+95% CI) Fitness = 47.3 + 0.000076 generation	Infinity
	7x0	(−95% CI) Fitness = 53.2 − 0.000058 generation	217,000
		(Mean) Fitness = 53.2 + 0.000060 generation	Infinity
		(+95% CI) Fitness = 53.2 + 0.000178 generation	Infinity
	9x0	(−95% CI) Fitness = 54.5 − 0.000179 generation	78,000
		(Mean) Fitness = 54.5 − 0.000021 generation	661,000
		(+95% CI) Fitness = 54.5 + 0.000137 generation	Infinity
	11x0	(−95% CI) Fitness = 54.7 − 0.000081 generation	175,000
		(Mean) Fitness = 54.7 + 0.000072 generation	Infinity
		(+95% CI) Fitness = 54.7 + 0.000225 generation	Infinity

**Table 2:** Paired *t*-test comparisons of average population fitness between deselections. Paired *t*-tests were used instead of one-way ANOVA as the former is targeted towards testing the difference in 2 sets of data; hence, paired *t*-test is a more appropriate test compared to one-way ANOVA. Our results show that there is no statistical difference between the average population fitness (from 25 replicates) from any 2 deselections, regardless of selection methods. However, the average population fitness from any deselection is significantly higher than control.

Paired *t*-test comparisons	*P* value
TS, loss-1 (generation 201 to 5200) versus loss-2 (generation 5401 to 10400)	0.617
TS, loss-1 (generation 201 to 5200) versus loss-3 (generation 10601 to 15400)	0.422
TS, loss-1 (generation 201 to 5200) versus loss-4 (generation 15801 to 20800)	0.656
TS, loss-2 (generation 5401 to 10400) versus loss-3 (generation 10601 to 15400)	0.061
TS, loss-2 (generation 5401 to 10400) versus loss-4 (generation 15801 to 20800)	0.683
TS, loss-3 (generation 10601 to 15400) versus loss-4 (generation 15801 to 20800)	0.158

FPS, loss-1 (generation 201 to 5200) versus loss-2 (generation 5401 to 10400)	0.980
FPS, loss-1 (generation 201 to 5200) versus loss-3 (generation 10601 to 15400)	0.975
FPS, loss-1 (generation 201 to 5200) versus loss-4 (generation 15801 to 20800)	0.483
FPS, loss-2 (generation 5401 to 10400) versus loss-3 (generation 10601 to 15400)	0.974
FPS, loss-2 (generation 5401 to 10400) versus loss-4 (generation 15801 to 20800)	0.522
FPS, loss-3 (generation 10601 to 15400) versus loss-4 (generation 15801 to 20800)	0.458

Loss-2 (generation 5401 to 10400), TS versus FPS	0.278
Loss-3 (generation 10601 to 15400), TS versus FPS	0.157
Loss-4 (generation 15801 to 20800), TS versus FPS	0.332

Control versus TS loss-2 (generation 5401 to 10400)	3.5 × 10^−17^
Control versus TS loss-3 (generation 10601 to 15400)	2.2 × 10^−17^
Control versus TS loss-4 (generation 15801 to 20800)	4.7 × 10^−17^
Control versus FPS loss-2 (generation 5401 to 10400)	1.7 × 10^−25^
Control versus FPS loss-3 (generation 10601 to 15400)	1.5 × 10^−25^
Control versus FPS loss-4 (generation 15801 to 20800)	6.1 × 10^−26^
